# Enhanced social learning of threat in adults with autism

**DOI:** 10.1186/s13229-020-00375-w

**Published:** 2020-09-22

**Authors:** Lisa Espinosa, Johan Lundin Kleberg, Björn Hofvander, Steve Berggren, Sven Bölte, Andreas Olsson

**Affiliations:** 1 Department of Clinical Neuroscience, Karolinska Institutet, Nobels väg 9, 171 65 Solna, Stockholm, Sweden; 2 Centre for Psychiatry Research, Department of Clinical Neuroscience, Karolinska Institutet, & Stockholm Health Care Services, Region Stockholm, Stockholm, Sweden; 3 Lund Clinical Research on Externalizing and Developmental Psychopathology (LU-CRED), Child and Adolescent Psychiatry, Department of Clinical Sciences Lund, Lund University, Lund, Sweden; 4 Division of Forensic Psychiatry, Region Skåne, Trelleborg, Sweden; 5 Center of Neurodevelopmental Disorders (KIND), Centre for Psychiatry Research, Department of Women’s and Children’s Health, Karolinska Institutet & Stockholm Health Care Services, Region Stockholm, Stockholm, Sweden; 6 Child and Adolescent Psychiatry, Stockholm Health Care Services, Region Stockholm, Stockholm, Sweden; 7 Curtin Autism Research Group, School of Occupational Therapy, Social Work and Speech Pathology, Curtin University, Perth, Western Australia

**Keywords:** Autism, Social fear learning, Vicarious threat, Eye tracking, Attention, Social cognition, Skin conductance, Anxiety

## Abstract

**Background:**

Recent theories have linked autism to challenges in prediction learning and social cognition. It is unknown, however, how autism affects learning about threats from others “demonstrators” through observation, which contains predictive learning based on social information. The aims of this study are therefore to investigate social fear learning in individual with autism spectrum disorder (ASD) and to examine whether typically developing social cognition is necessary for successful observational learning.

**Methods:**

Adults with ASD (*n* = 23) and neurotypical controls (*n* = 25) completed a social fear learning (SFL) procedure in which participants watched a “demonstrator” receiving electrical shocks in conjunction with a previously neutral conditioned stimulus (CS+), but never with a safe control stimulus (CS−). Skin conductance was used to measure autonomic responses of learned threat responses to the CS+ versus CS−. Visual attention was measured during learning using eye tracking. To establish a non-social learning baseline, each participant also underwent a test of Pavlovian conditioning.

**Results:**

During learning, individuals with ASD attended less to the demonstrator’s face, and when later tested, displayed stronger observational, but not Pavlovian, autonomic indices of learning (skin conductance) compared to controls. In controls, both higher levels of attention to the demonstrator’s face and trait empathy predicted diminished expressions of learning during test.

**Limitations:**

The relatively small sample size of this study and the typical IQ range of the ASD group limit the generalizability of our findings to individuals with ASD in the average intellectual ability range.

**Conclusions:**

The enhanced social threat learning in individuals with ASD may be linked to difficulties using visual attention and mental state attributions to downregulate their emotion.

Autism spectrum disorder (ASD) is a neurodevelopmental condition characterized by a wide range of alterations in social cognitive and affective processes (American Psychiatric Association 2013), including face processing [1–3], cognitive empathy [4], and social attention [5]. Between 40 and 70% of individuals with ASD also fulfill the criteria for one or more anxiety disorder [6, 7] or highly idiosyncratic fears and aversive reactions to sensory input [8, 9]. Recent research has documented that individuals with ASD have difficulties forming stable predictions about future events, leading to impaired learning about relationships between events in the world, so called “predictive learning” [10–13]. Because knowledge about others’ mental states and emotions is often uncertain and probabilistic, learning that involves social information may be particularly challenging for autistic individuals [13–16]. Atypical prediction learning could therefore be a common mechanism in ASD, underlying both social cognition alterations, repetitive behaviors, fears, and aversive reactions to sensory stimuli [10–12].

The current study had two goals: to extend our knowledge about social prediction learning in ASD, and to determine whether typically developed social cognition is necessary for successful social fear learning (SFL). Addressing the first goal, we examined the learning of threat responses and fear by means of social transmission through observation, here referred to as SFL, in a sample of individuals with ASD and neurotypical controls. SFL has served as an experimental model to explain the development of anxiety disorders [17–19] and is defined as the process through which a predictive association between a stimulus and an aversive outcome is acquired through social means in the absence of direct experiences with the aversive stimulus, for example by observing another individual’s expressions of distress to an aversive stimulus [20]. SFL depends on brain circuits involved in learning threat responses and fear through direct, personal experiences (traditional Pavlovian conditioning), including the amygdala and hippocampus, in concert with cortical regions involved in social cognition and action understanding [21]. Facial expressions of distress may have inherent aversive properties, therefore augmenting SFL [22, 23]. By enhancing the salience of distress cues in a demonstrator, SFL may contribute to socialization and the transmission of important information between individuals [18, 24]. Although SFL is adaptive at a species level, it can also lead to learning of maladaptive fears by observation of others, thereby potentially contributing to the etiology of anxiety disorders. For example, several studies have shown that children can learn fear and avoidance behaviors to previously non-aversive stimuli by observation of parents of peers [18, 19, 25]. These associative mechanisms in fact partially overlap with those underlying learning through direct, personal experiences (e.g., Pavlovian and instrumental conditioning [26]) and are supported by research on both the neural and computational bases of SFL [26].

Our second goal was to determine whether typically developed social cognition was necessary for successful SFL. The role of social cognition in SFL is largely unknown. Previous studies in neurotypical populations have shown that perspective taking (cognitive empathy) can enhance social learning [22], by enhancing attention to emotionally relevant stimuli [27], such as faces [23]. Using eye-tracking technology, an earlier study [28] demonstrated that the distribution of visual attention during SFL predicted the strength of the subsequent threat response. The brain mechanisms implicated in SFL are also overlapping with those related to social cognition [21]. These findings suggest that social cognitive ability underlies typical SFL. If this is correct, individuals with ASD would be expected to show *reduced* SFL compared to neurotypical individuals. However, social cognitive abilities may also help individuals to downregulate aversive responses through reappraisal [29], i.e., by changing their interpretation of the emotional stimuli and therefore affecting their emotional reaction [30]. This suggests that social cognitive challenges in ASD may instead lead to *enhanced* SFL. The same conclusion would be supported by previous findings that an impaired ability to predict the demonstrator’s response to the shock during SFL augments the vicarious response, leading to stronger learning [31]. If ASD is associated with enhanced SFL, this would point to a potential mechanism underlying the increased prevalence of fears and aversive reactions to sensory input in this population [8, 9]. Conversely, reduced SFL in ASD could be a mechanism underlying difficulties in social interactions.

We examined SFL in adults with ASD in the typical IQ range. Since both reduced and enhanced SFL could be predicted based on previous studies, we did not direct the hypothesis. Attention has been implicated as a factor mediating the relationship between social cognition and learning [23, 27], and measured eye movements were therefore recoded during learning.

Although no previous studies have examined SFL in individuals with ASD, a small number of studies suggest that autonomic indices of direct fear learning through Pavlovian conditioning are largely intact in this population [32–34], but reduced differentiation between threat and safety cues in amygdala activity has also been reported [35]. One study reported stronger SFL in neurotypical individuals with high levels of autistic traits [36]. To replicate previous research on Pavlovian conditioning, and to enable its direct comparison with SFL, each participant underwent both Pavlovian and observational learning.

## Methods

### Participants

Individuals with a diagnosis of ASD (*n* = 23; 14 females) were recruited through autism interest and self-advocacy organizations. Diagnoses were corroborated by a clinical psychologist using the Autism Diagnostic Observation Schedule-Second Edition (ADOS-2) [37]. A neurotypical control group (*n =* 25; 9 females) was recruited through poster advertisement at the Karolinska Institutet campus (see Table 1 for an overview of demographic and trait information). Psychological assessment of both groups was carried out using the Autism Quotient [38] as a screening measure for autistic traits, State-Trait Anxiety Inventory [39] to measure trait anxiety, and the empathy quotient [40] to assess trait empathy. IQ was measured using the WAIS-IV [41]. Participants in the control group reported no current or previous psychiatric disorder, including ASD, and screened negative for ASD on the Autism Quotient [42] (AQ; all scores < 17). Apart from the participants included in the analysis, two individuals recruited for the ASD were excluded because they did not reach diagnostic threshold on the ADOS-2, and three participants were excluded from the control group because of a self-reported diagnosis of borderline personality disorder, current major depression, or because of data loss due to equipment failure. Participants with valid eye-tracking data from less than two events per condition (ASD: *n =* 6; control: *n =* 4) were excluded from analyses involving these measures. As expected, ASD participants had higher levels of self-reported autistic traits on the AQ as well as anxiety on the State-Trait Anxiety Inventory (STAI) [39]. Groups did not differ in IQ as measured by the General Ability Index (GAI) of the Wechsler Adult Intelligence Scale (WAIS-IV [41];) or sex ratio, although the ASD group was slightly older (see Table 1). In the ASD group, 15 participants were on psychoactive medication. The types of medication were psychostimulants (methylphenidate, *n* = 4; dexamphetamine, *n* = 1), non-stimulant ADHD medication (atomoxetine, *n* = 1), anxiolytics (hydroxyzine, *n* = 3; alimemazine, *n* = 1), selective serotonin reuptake inhibitors (fluoxetine, *n* = 1; sertraline, *n* = 4), and selective serotonin and noradrenaline reuptake inhibitors (venlafaxine, *n* = 1; mirtazapine *n* = 1). In a preliminary analysis, we compared the ASD participants with and without psychoactive medication on all dependent variables used in the analyses and found no group differences, why they were pooled for the main analysis.

**Table 1:** Demographic and sample characteristics

Measures	ASD		Control		*p*-values
	M	(SD)	M	(SD)	
Age	31.61	(9.50)	26	(4.90)	.012
Gender (female/male)	14/9		9/16		.148
IQ	108.7	(20.13)	111.2	(19.00)	.66
EQ	25.52	(9.92)	49.4	(9.99)	<.001
AQ	31.13	(8.60)	11.88	(5.92)	<.001
STAI-T	50.67	(7.81)	36.04	(7.95)	<.001
ADOS-2 (total score)	20.08	(4.68)	_		_

*IQ* General Ability Index (GAI) of the WAIS-IV, *EQ* Empathy Quotient, *AQ* Autism Quotient, *STAI-T* STAI-T State-Trait Anxiety Inventory, Trait Scale, *ADOS-2* Autism Diagnostic Observation Schedule-Second Edition

### Experimental protocol

**Fig. 1:**
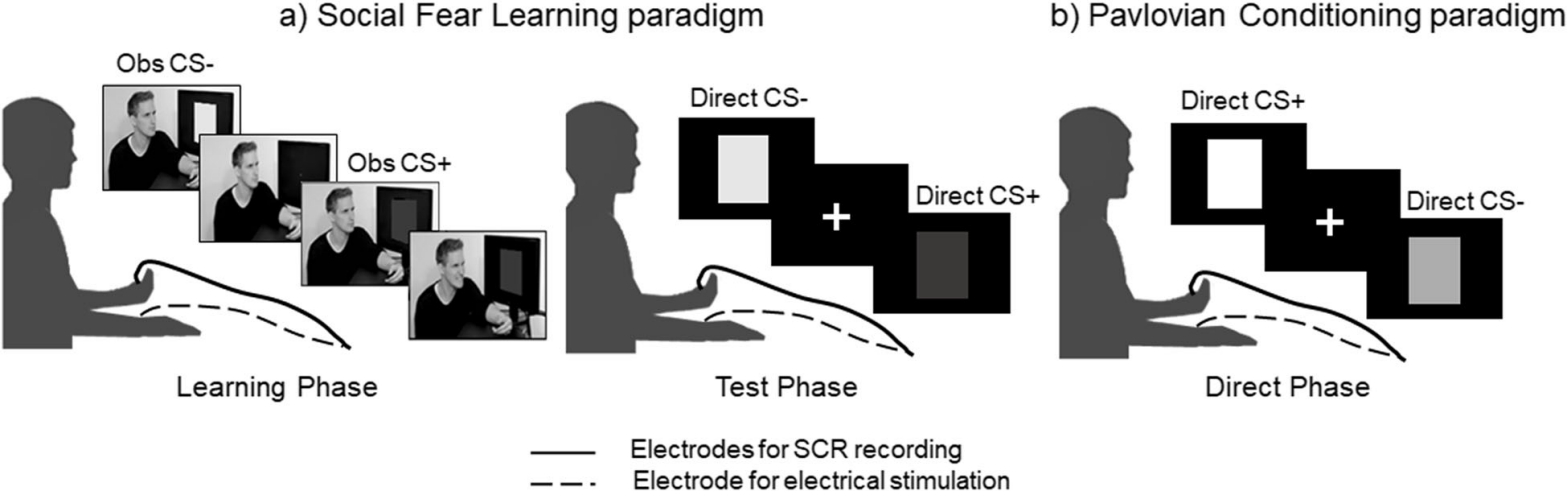
Schematic overview of the experimental procedure. **a** Social fear learning (SFL) paradigm, where the participant observes, on a computer screen, a demonstrator receiving shocks at one of the picture stimuli (CS+) but not at the other (CS−) (learning phase), followed by a test phase where the participant is directly exposed to the same picture stimuli, without receiving any shock. **b** The Pavlovian conditioning paradigm, where the participant is directly exposed to (new) picture stimuli and received shocks at CS+ but not at CS−

The SFL procedure was adapted from previous studies [28, 43] and is illustrated in Fig. 1. During an initial *learning phase*, participants viewed a video of a male individual (henceforth the demonstrator) facing a computer screen on which colored squares were sequentially presented. The demonstrator received an electric shock when one color (CS+), but never when the other color (CS−), was presented on a screen in front of him. Participants were told that they would go through the same experiment as the demonstrator. Six CS+ and 6 CS− were presented one at a time in one of two possible presentation orders, counterbalanced between participants, with a duration of 6000 ms and an inter-stimulus interval with a mean of 13.7 s (SD = 1.12 s). Which colored square was CS+ and which was CS− was also counterbalanced between participants. Importantly, no shocks were administered to the participants, meaning that learning was only possible through observation. The second phase of the SFL procedure (henceforth *the test phase*) started directly after the participants watched the movie. Participants were informed that they would now participate to a similar experiment and reminded that the same stimulus would be paired with electric shocks. During this phase, the CS+ and CS− were presented six times one at a time in pseudorandom order, with a stimulus duration of 6000 ms.

A Pavlovian conditioning procedure was completed directly after the main experiment. During this phase, mild electrical shocks were delivered. The electric shock stimulus was a monopolar 100 ms DC-pulse electric stimulation (STM200; BIOPAC Systems Inc) applied to the participant’s right forearm. Prior to Pavlovian conditioning, participants themselves adjusted the level of electrical shocks to be uncomfortable, but not painful. During Pavlovian conditioning, the CS+ and CS− were colors previously not used in the SFL procedure. The Pavlovian conditioning phase included the same number of trials and presentation sequence, with the exception that the CS+ and CS− were always shown in isolation (i.e., no demonstrator) and that shocks were delivered directly to the participant during learning.

### Data recording

Eye movements were recorded with a corneal reflection eye tracker (iView X RED, Sensomotoric Systems GmbH) at a sample rate of 50 Hz. Participants were seated at approximately 70 cm distance from a 24″ monitor. All participants completed a 13 point operator-controlled calibration procedure at the beginning and before each part of the experiment. Skin conductance data were recorded at a sample rate of 250 Hz with a BIOPAC MP100A system at palmar sites of the participants’ left hand.

### Data analysis

Gaze and SCR data were analyzed during the 0–5500 ms interval after the onset of the CS+ and CS− (henceforth CS+ and CS− events). We also defined a shock interval (0–3000 ms after shock delivery to the demonstrator) and a corresponding no shock interval during CS presentations. Skin conductance was analyzed offline with the AcqKnowledge software (BIOPAC, CA). The raw skin conductance signal was low-passed filtered (threshold of 1 Hz) and high-passed filtered (threshold of 0.01 Hz). The SCR amplitude was defined as the largest peak-to-peak value in microsiemens (μS) during the 500–4500 ms time window after the onset of each event. Responses with amplitudes smaller than 0.02 μS were coded as 0. The raw data values were square root transformed to approach normal distribution. In the observation learning phase, the first trial of each CS in each part was excluded because no differential learning could occur until the first CS shock pairing was presented. The CR was operationalized, for each part, as the average SCR amplitude to the CS+ minus the average SCR amplitude to the CS−.

**Fig. 2:**
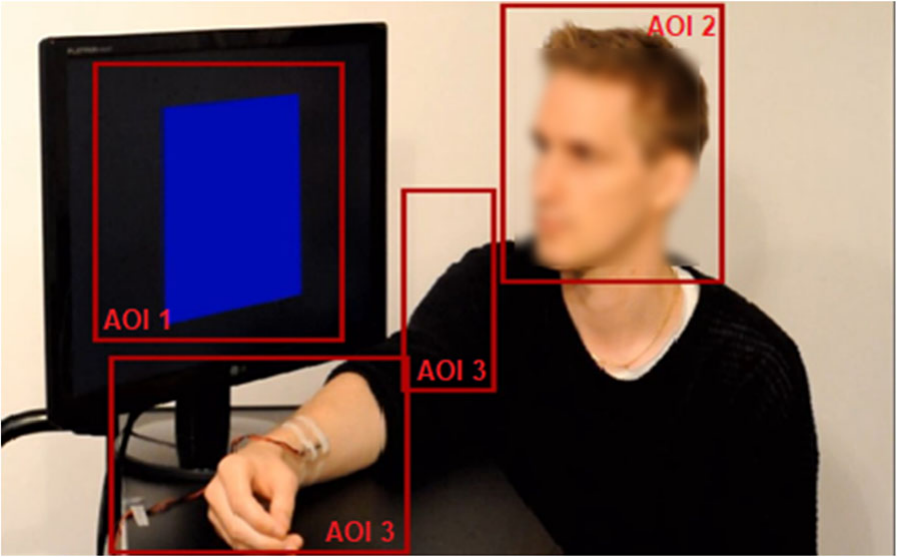
Illustration of the stimuli presented on the computer screen in the social fear learning task with areas of interest (AOIs) used in the eye-tracking analysis in red. AOI 1 = the demonstrator’s face, AOI 2 = the CS+/− that was presented to the demonstrator, AOI 3 = the demonstrator’s arm and shoulder. Note that the demonstrator’s face is only blurred here to maintain anonymity

Fixations were identified with a dispersion-based filter using the IDF Event Detector Software (Senso-Motoric Instruments GmbH). The minimum fixation duration was set to 100 ms, and the dispersion diameter was set to cover approximately 1° of the visual field. Further analyses were conducted with custom scripts written in MATLAB (Mathworks, Inc.). Rectangular areas of interest (AOIs) covering the following areas were defined (Fig. 2): (1) the demonstrator’s face, (2) the CS+/− that was presented to the demonstrator, (3) the demonstrator’s arm and shoulder, including the shock electrode. Data was normalized to the total fixation time at the screen.

All eye-tracking data from an event were discarded if it contained less than 15% valid fixation time at the screen. Since many participants lacked eye-tracking data from one or more trials, eye tracking data were averaged over all valid events. The groups did not differ in average number of valid events (see Table 2). All fixation time variables were square root transformed to approach normal distribution.

**Table 2:** Average number of valid events in the eye-tracking analysis in the ASD and control groups

Condition	ASD		Control	
	***M***	**(SD)**	**M**	**(SD)**
CS+	4.5	(1)	4.65	(0.8)
CS-	4.65	(0.8)	4.35	(1.14)
Vic shock	3.45	(0.76)	3.65	(0.75)
No shock	5	(1.21)	5.45	(1.15)

### Statistical analyses

Statistical analyses were conducted in PASW Statistics (IBM Corporation). General linear models (GLM) were used to compare groups and experimental conditions and to analyze relationships between eye gaze and skin conductance response (SCR) data. Significance level was set to .05. Significant interaction effects were followed up with *t* tests. Follow-up multiple comparisons were corrected using Bonferroni procedure. Independent samples *t* tests were used to compare groups on demographical and clinical background variables (see Table 1).

## Results

Below, we first present the results (SCR and eye-tracking) from the learning and test phases of the SFL experiment. This is followed by a description of the relationship between SFL and trait measures. Finally, we describe the results (SCR) from the Pavlovian conditioning experiment.

### Learning phase: responses to the CS+ and CS−

A repeated measures GLM with CS type (CS+, CS−) and trial (1–6) as within subjects factors and diagnostic group (ASD, controls) as between-subjects factor showed a main effect of CS type (*F*(1, 46) = 14.56, *p* < .001, *η*^2^ = .24), reflecting higher SCRs to the CS+ than the CS− (i.e., a conditioned response). There was also a main effect of trial reflecting decreasing SCRs during the course of the experiment (*F*(1, 46) = 5.06, *p* < .001, *η*^2^ = .12), but no main effect of group (*F*(1, 46) = 2.60, *p* = .11, *η*^*2*^ = .05) and no two- or three-way interaction effects involving group, CS type, and trial (all *p > .*25). To sum up, these results showed that both ASD and control individuals acquired a threat response during the learning phase.

### Learning phase: responses to vicarious shocks

A main effect of event type (vicarious shock, vicarious no shock) was found (*F*(1, 46) = 77.58, *p* < .001, *η*^2^ = .63), driven by higher SCRs during vicarious shock intervals than vicarious no shock intervals. There was also a significant main effect of trial (*F*(3, 46) = 17.98, *p* < .001, *η*^2^ = .38), reflecting decreased SCRs during later trials, and a marginally significant interaction between event type and trial (*F*(3, 46) = 2.70, *p* = .057, *η*^2^ = .16). No main effect of group *F*(1, 46) = 1.984, *p* = .17, η^2^ = .04 or event type × group interactions were found *F*(1, 46) = 2.64, *p* = .11, *η*^2^ = .05). These results showed that both individuals with ASD and controls had a stronger SCR to seeing the demonstrator receiving shocks as compared to not receiving shocks.

### Learning phase: eye movements during CS+/CS− events

Analyses of the fixation time at the demonstrator’s face showed a main effect of group, *F*(1, 37) = 4.54; *p* = .040, *η*^2^ = .11, reflecting longer fixation time at the face in the control group, and a significant group × condition interaction, *F*(1, 37) = 4.29, *p* = .045, *η*^2^ = .10. Follow-up comparisons using Bonferroni correction showed that the ASD group fixated less at the face during CS+ events (*M* = .65, SD = .19) compared to the control group (*M* = .41, SD = .31) (*t* (38) = 2.91; *p* = .024, *d* = 0.94) but not during CS- events, *t* (38) = 1.13; *p* = .26, *d* = 0.23. Fixation time during CS+ events was not linked to anxiety score (STAI-T score) for either of the groups (control group: *r*(20) = .12, *p* = .61; ASD group, *r*(20) = .33, *p* = .15). No main effect of condition was found, *F*(1, 38) = < 0.01, *p* > .25.

Analyses of the fixation time at CS AOI showed a main effect of condition, *F*(1, 37) *=* 6.24, *p* = .012, *η*^2^ = .14), reflecting shorter fixation times at the CS AOI during CS+ events. No significant main effect of group, *F*(1, 37) = 2.10, *p* = .16, *η*^2^ = .05, or group × condition interaction, *F*(1, 37) = 2.70, *p =* .11, *η*^2^ = .07, was found. The analyses of the fixation time at arm/shoulder AOI showed no main or interaction effects (all *ps* > .35), indicating that both groups looked similarly at this area across conditions. Furthermore, no relationship between the trait measures and SFL was found for any of the groups.

### Learning phase: eye movements during shock and no shock intervals

Fixation time at the models face increased during shock trials as compared to no shock trials, reflected in a marginally significant main effect of condition, *F*(1, 37) = 3.98, *p* = .053, *η*^2^ = .10. Neither the main effect of group, *F*(1, 37) = 0.89, *p* > .25, *η*^2^ = .02, nor the group × condition interaction, *F*(1, 37) = 0.26, *p >* .25, *η*^2^ = .01, was significant.

Fixation time at the CS AOI decreased during shock intervals compared to no shock intervals (*F*(1, 37) = 38.46, *p* < .001, *η*^2^ = .54). The main effect of group was not significant for fixation time at the CS AOI, *F*(1, 37) = 2.55, *p =* .14, *η*^2^ = .06, but a group × condition interaction was found (*F*(1, 37) *=* 9.34, *p* = .004, *η*^2^ = .20).

Follow-up comparisons using Bonferroni correction showed that controls directed less attention to the CS AOI during shock intervals (*M* = .26, SD = .24) as compared to no shock intervals (*M* = .59, SD = .19), *t* (20) = 6.59, *p* < .00, *d* = 1.57, whereas the difference between conditions was not significant in individuals with ASD, *t* (19) = 2.31, *p* = .13, *d* = 0.44.

During no shock intervals, the ASD group looked less at the CS AOI (*M* = .39, SD = .25) than controls (*M* = .59, SD = .19), *t* (38) = 2.95, *p =* .02, *d* = 0.94, whereas no group difference was found in fixation time at the CS AOI during vicarious shock intervals, *t* (38) = 0.18, *p >* .25, *d* = 0.08. No main or interaction effect was found for the fixation time at arms and shoulder during shock intervals (all *ps* > .25).

### Test phase

**Fig. 3:**
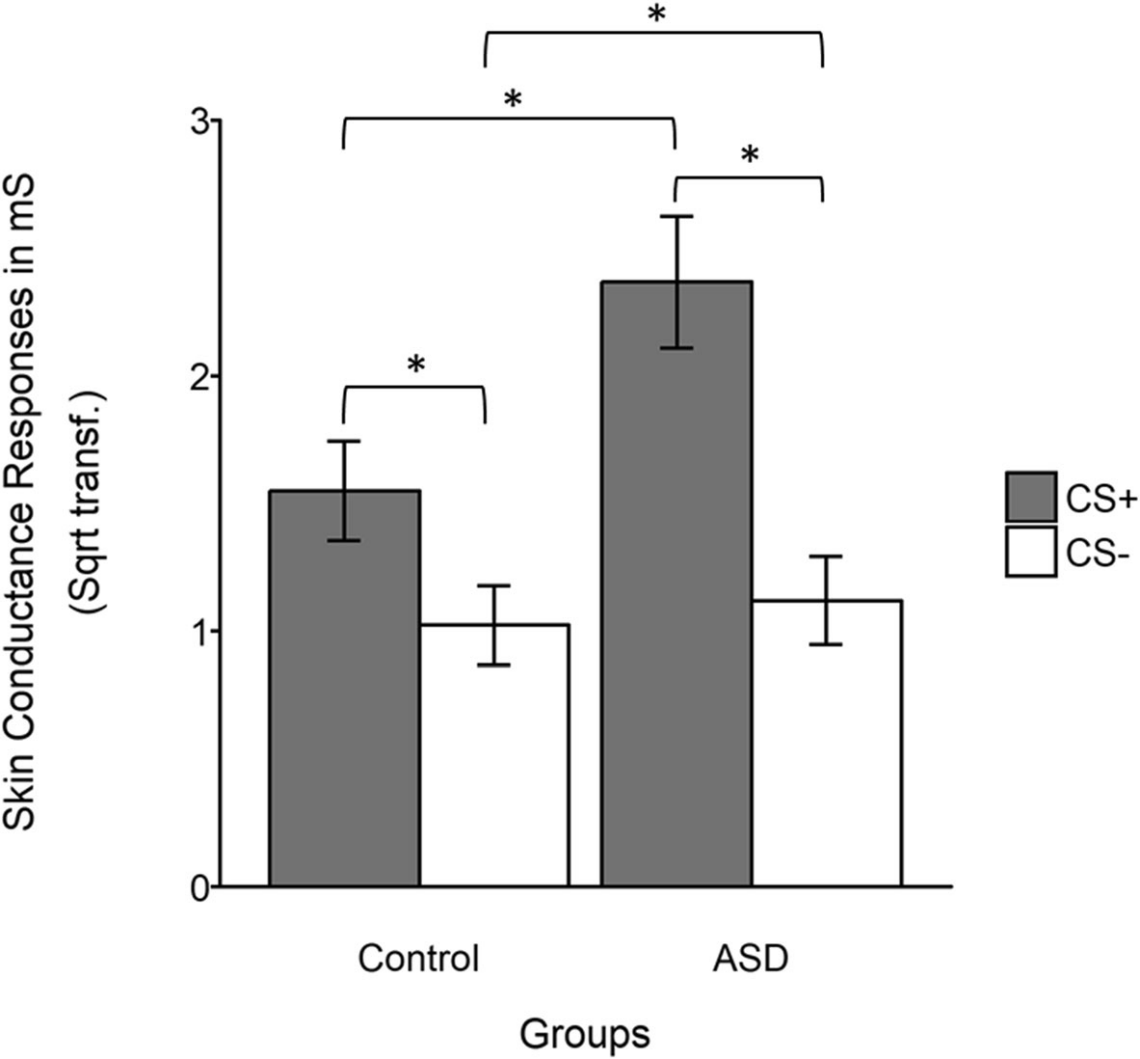
Average skin conductance responses during CS+ and CS− events in ASD and control participants. This figure shows an interaction effect between group and CS, indicated by larger responses to the CS+ in the ASD group, but no group difference during CS- trials. *p < .05. Error bars cover means +/− 1SEM.

SCR amplitude was larger during CS+ than CS− trials, *F*(1, 46) = 31.50, *p* < .001, *η*^*2*^ = .41. There was also a main effect of trial, reflecting that the SCR amplitude decreased over the course of the test phase (*F*(1, 46) = 13.83, *p* < .001, *η*^*2*^ = .45), and a CS × trial interaction, reflecting that this decrease was larger during CS+ trials, (*F*(5, 46) = 2.695, *p* = .024, *η*^*2*^ = .10). To sum up, our results show that a conditioned response (CR) was maintained during the test phase, but that responses to the CS+ decreased after presentations without shocks (i.e., extinction). Importantly, as illustrated in Fig. 3, an interaction effect between group and CS was also found (*F*(1, 46) = 5.20, *p* = .024, *η*^*2*^ = .10), displaying larger responses to the CS+ in the ASD group (*F*(1, 47) = 6.74, *p =* .014, *η*^*2*^ = .12), but no group difference during CS trials (*F*(1, 47) = 0.68, *p = .*25, *η*^*2*^ = .01). No significant relationships between the SCR to CS+ and anxiety scores (STAI score) were found (control group: *r*(25) = − .07, *p* = .73; ASD group, *r*(23) = − .26, *p* = .22).

### Relationships between eye movements, trait empathy, and the conditioned response

A GLM with the CR as dependent variable was used to test independent contributions of fixation time at the face and trait empathy. In controls, both fixation time at the face during CS+, *F*(1, 16) = 8.21, *p =* .011, *η*^*2*^ = .34, and trait empathy, *F*(1, 16) = 5.58, *p =* 0.31, *η*^*2*^ = .26, independently predicted lower CR. No relationship between these measures was found in the ASD group (all *p >* .25).

### Pavlovian fear learning

A CR was found during acquisition, reflected in higher SCR on CS+ than CS− trials, *F*(1, 46) = 21.87; *p* < .001; *η*^2^ = .33. No main effect of group (*F*(1, 45) = 0.94, *p* > .25; *η*^2^ = .02) or group × CS interaction were found, *F*(1, 46) = 2.61, *p* = .11, *η*^*2*^ = .06. As expected, CR amplitude decreased during later trials of acquisition, *F*(1, 46) = 22.30, *p* < .001, *η*^*2*^ = .33, but this effect was not qualified by two- or three-way interactions involving group (all *p > .25*). The CR was maintained during extinction, *F*(1, 46) = 12.62, *p* = .001, *η*^*2*^ = .22, but there were no main effects of group, *F*(1, 45) = 1.59, *p* = .224; *η*^*2*^ = .033, or interaction between group and CS, *F*(1, 45) = 1.43; *p =* 0.239, *η*^*2*^ = 0.03. Conditioned response amplitude decreased during the course of extinction, *F*(1, 45) = 5.04; *p* = .001, *η*^*2*^ = .10, but this effect was not qualified by two- or three-way interactions involving group (all *p > .25*). To sum up, the ASD group showed a typical pattern of Pavlovian fear learning.

## Discussion

We examined threat learning through social (observational) and Pavlovian learning paradigm in individuals with ASD and neurotypical controls. Our results showed that in comparison to controls, individuals with ASD displayed stronger learned defensive responses when learning was transmitted solely by observation and without any personal experiences of the aversive event. Eye-tracking analyses showed that individuals with ASD attended less than controls to the demonstrator’s face in the presence of a threat cue (the CS+), supporting earlier reports of reduced social attention [5, 44, 45] and atypical face-scanning patterns [46] in this group. For controls, the fixation time at the demonstrator’s face during learning was inversely related to the strength of SFL, while no relationship between attention and learning was found in the ASD group. This suggests that typically developed individuals attend to a demonstrator’s facial expressions during vicarious learning situation as a means to evaluate the threat value of the predictor (here the CS+) and modulate learning. This process may be impaired in individuals with ASD. It is possible that attention to the face in control participants facilitated a downregulation of the emotional response triggered by the demonstrator’s distress. This conjecture was supported by our finding that higher levels of trait empathy (i.e., social cognitive ability) were related to weaker expressions of SFL. Taken together, this suggests that enhanced SFL in the ASD group may have resulted from a reduced ability to use facial information to downregulate the emotional response. However, since our data are correlational, other interpretations are also possible. Our results add to the inconsistent findings in previous research investigating physiological reactivity to threat-related facial expressions in autism [e.g., 46–48, 50]. For example, one previous study reported that in contrast to typically developing peers, children with ASD did not differentiate between stimuli previously coupled with fearful or happy facial expressions in their pupil dilation response [47]. The difference between this study and the present results may be related to age of the samples (adults vs. preschoolers), physiological measures (SCR vs. pupil dilation), and experimental tasks.

Intolerance of uncertainty has been suggested as one of the underlying mechanisms of anxiety in ASD [e.g., [9]]. We used a probabilistic reinforcement schedule, in which the CS+ was followed by a shock in 75% of the trials during learning, and this degree of uncertainty may have potentiated SFL in the ASD group. Finally, it is possible that the pattern of reduced social attention during CS+ presentations was caused by enhanced arousal rather than contributing to it. Previous studies have suggested that reduced social attention in ASD may be a means of reducing arousal and should therefore be most likely in high emotional states [48].

In addition to providing, for the first time, a window into the combined attentional and autonomic responses underlying SFL in individuals with ASD, our results indicate that because the social cognitive difficulties in the range experienced by participants with ASD in this study did not prevent SFL, a typical social cognitive ability might not be necessary for this form of learning to occur. This does of course not mean that social cognition does not contribute to SFL. Indeed, ample research has demonstrated that social cognition can contribute in a range of ways to SFL [26]. Although it is important to recognize that ASD is a heterogeneous condition, and the individuals in our study were likely to vary in their social cognitive capacity, we did not detect any relationships between threat learning and the AQ in the ASD group. This argues against a simple interpretation of the role of social cognition in observational threat learning.

Importantly, individuals with ASD showed highly typical fear learning in the Pavlovian conditioning task, suggesting that enhanced fear learning was relatively specific for social learning. This replicated previous research and extended it into the realm of social learning.

Individuals with ASD have an increased risk for classical anxiety disorders recognized in the DSM-5, as well as for idiosyncratic fears [e.g., [8, 9]]. Enhanced SFL could contribute to the development of these problems, particularly in individuals experiencing difficulties with interpreting others’ reactions and emotions.

### Limitations

Since the relationship between eye movements and SFL was merely correlational, the conclusions that can be drawn about causality are limited. Another limitation of this study is related to the relatively small sample size. ASD is a heterogeneous condition, and the current study was not statistically powered to identify potential subgroups. Replications are therefore needed. Because our research question focused on the SFL, the Pavlovian learning phase always succeeded the SFL procedure. This means that we cannot exclude the possibility that prior experience from SFL influenced subsequent Pavlovian learning. However, a recent study investigated both SFL and Pavlovian learning in neurotypical participants, while counterbalancing their order and showed no effect of order [49]. This can suggest that the order of completion in the current study might not have a major impact on the results of Pavlovian learning. Furthermore, in the current study, individuals with ASD and controls did not differ in IQ, which strengthens the match between the groups. At the same time, the relatively high IQ in the ASD group limits the generalizability of our findings to individuals with ASD in the average intellectual ability range. Finally, individuals with ASD and controls were not matched on levels of anxiety, limiting the specific conclusions that can be drawn about ASD and comorbid anxiety. Importantly, and arguing against that this posed a problem in our study, no links between anxiety scores and the main SLF findings were found. An important venue for future studies would be to compare SFL in populations with ASD with and without enhanced levels of fear and anxiety.

## Conclusions

To sum up, we found evidence for enhanced social learning of fear in individuals suffering from ASD. This is especially interesting because SFL is believed to play an important role in the development of anxiety disorders, which are more common in individuals with ASD as compared to the general population. Our results showed that in normally developed individuals, but not those with ASD, attention to the demonstrator’s face was linked to a subsequent reduction of the learned fear response. These findings suggest that individuals with ASD lack the ability to capitalize on facial information in the social context to aid the regulation of anxious responses, providing further clues towards understanding why this population more often suffers from problems with anxiety, and adding to the growing literature investigating learning from observing others [13–15, 48] and responsiveness to distress in others [50] in individuals with ASD.

More research is needed to understand the underlying mechanisms of these putative processes. Finally, our results also suggest that typical social cognitive ability is not necessary for a successfully acquired SFL, implying that there are many routes to acquire and express socially learned emotional information.

## Data Availability

The datasets used and/or analyzed during the current study are available from the corresponding author upon request.

## Abbreviations

ASD: Autism spectrum disorder
SFL: Social fear learning
ADOS-2: Autism Diagnostic Observation Schedule-Second Edition
AQ: Autism Quotient scale
STAI: State-Trait Anxiety Inventory
WAIS-IV: Wechsler Adult Intelligence Scale
CS+: Conditioned stimuli followed by an electrical stimulation
CS−: Conditioned stimuli never followed by an electrical stimulation
CR: Conditioned response(s)
SCR: Skin conductance response
